# Trends of infant vaccination timeliness and completion in selected urban slum communities in Ibadan, Southwestern Nigeria: A four-year review

**DOI:** 10.1371/journal.pone.0285805

**Published:** 2023-05-23

**Authors:** Folusho Mubowale Balogun, Eniola Adetola Bamgboye, Adebola Emmanuel Orimadegun

**Affiliations:** 1 Institute of Child Health, College of Medicine, University of Ibadan, Ibadan, Nigeria; 2 Institute of Child Health, University College Hospital, Ibadan, Nigeria; 3 Faculty of Public Health, Department of Epidemiology and Medical Statistics, College of Medicine, University of Ibadan, Ibadan, Nigeria; Ladoke Akintola University of Technology Teaching Hospital: LAUTECH Teaching Hospital, NIGERIA

## Abstract

**Background:**

Suboptimal infant vaccination is common in Nigeria and multiple interventions have been deployed to address the situation. Child health indicators are reported to be worse in urban slums compared with other urban areas, but urban data are usually not disaggregated to show these disparities. Examining the timeliness and completion of infant vaccination in urban slums is important to determine the effectiveness of existing interventions in improving infant vaccination among this vulnerable population. This study explored the trends of infant vaccination in selected urban slum communities in Ibadan, Southwest Nigeria between November 2014 and October 2018.

**Methods:**

This was a cross sectional study where infant vaccination data were extracted from the immunization clinic records of six primary health care centers that were providing infant vaccination services for seven urban slum communities. Data was analyzed using descriptive statistics and Chi square test at α = 05.

**Results:**

A total of 5,934 infants vaccination records were reviewed, 2,895 (48.8%) were for female infants and 3,002(50.6%) were from Muslim families. Overall, only 0.6% infants had both timely and complete vaccination during the four years under study. The highest number of infants with timely and complete vaccination were seen in 2015(12.2%) and least in 2018(2.9%). Regarding timeliness of the vaccines, BCG, was the least timely among the vaccines given at birth and the pentavalent and oral polio vaccines’ timeliness reduced as the age of the infants increased. Both yellow fever and measles vaccines were timelier than the pentavalent vaccines. Vaccines were most timely in 2016(31.3%) and least timely in 2018(12.1%). Those from Muslim families significantly had delayed and incomplete vaccinations compared with those from Chrisitan families (p = 0.026).

**Conclusion:**

Infant vaccinations were significantly delayed and incomplete in the study communities during the years reviewed. More focused interventions are required to ensure optimal vaccination of the infants.

## Introduction

Nigeria has had one of the highest proportions of nonimmunized infants in the world over the last decade, and this trend is cause for concern given the country’s high mortality rate for children under the age of five [1]. From 2008 to 2018, the percentage of children who did not receive any of the required vaccines decreased from 29% to 19% [2]. While this trend was promising, progress toward immunization coverage was inadequate to meet the Sustainable Development Goal 3 target of 90% coverage for all necessary vaccinations for children aged 12 to 23 months [3]. Several factors have been identified as reasons for this poor uptake of infant immunization including poor infrastructure for cold chain and distribution of vaccines [4] mistrust of the government, rumors of HIV contaminated vaccines and contraceptives (as a tool to control population growth) [5,6], poor health care staff attitude [6], and unchallenged parents’ attitudes [7].

The Nigeria National Demographic Health Surveys have consistently shown that nonimmunized infants in Nigeria are more in the rural areas than urban and found in the households with the least wealth quintile than others [8,9]. This is an indication that socioeconomically disadvantaged communities such as those living in the slums in urban areas are likely to have poor immunization coverage. Specifically, earlier reports have suggested that infant immunization uptake in urban slums may be worse than what obtains in rural areas [10,11]. Whereas attention have been focused on improving immunization uptake in rural areas, the urban slums are usually neglected because their immunization uptake rates are subsumed by the high coverage of the whole urban communities, masking their peculiarities. Several research from developing countries have shown that the disadvantaged subpopulation of large city metropolitan areas have the least access to healthcare due to severe poverty, lack of knowledge and social exclusion. It is likely that the health coverage of the urban slums in Nigeria would exhibit a similar pattern.

For an effective infant immunization, both the timeliness of the vaccines and the completion of the vaccination courses are important [12,13]. It is important that all infants complete their immunization schedule because this ensures protection from vaccine preventable diseases as well as herd immunity for these diseases. However, if the vaccines are not given as at when due, these benefits of completion of immunization are undermined and the infants become vulnerable to vaccine preventable diseases (VPDs) during the window of delayed vaccination. At the same time, the infants with delayed vaccination will be a threat to other children because they can transmit infections to healthy children [14]. Delayed vaccination is also a threat to herd immunity as it will not be realized or the already established ones will be lost, thereby increasing the likelihood of an epidemic.

Many studies have reported immunization coverage in Nigeria, but few have given attention to timeliness of vaccines. A recent systematic review on timeliness of vaccination identified four studies from Nigeria with relevant data [15] and the four studies were conducted in urban areas [16–19]. Sadoh and colleagues [16] examined the hospital records of a government owned immunization clinic in Benin, and reported that the timeliness for each of the vaccines ranged from 18.7% to 61.5% and delays occurred in 18.9% and 65.0% for different vaccines. Another study in the same city reported 51.7% immunization timeliness among 174 infants in a private clinic [17]. The higher timeliness observed in the later than former study in the same urban area may be as a result the differences in the setting. Infants’ parents pay for consumables in private hospitals, while immunization is almost entirely free in public health facilities, giving them a sense of responsibility to ensuring vaccine timeliness. Odusanya reported a lower vaccine timeliness rate of 26% among 110 children aged 12 to 23 months from a rural Nigerian setting [18]. However, none of the four Nigeria studies included in the systematic review by Masters and colleagues specifically focused on the people living in the urban slums [20].

The drivers of poor access and usage of health services such as vaccination vary with diverse local contexts within and across countries [15]. Therefore, to ensure universal health coverage, the identification of unique barriers to community access to healthcare is crucial. In Nigeria, the timeliness and completion of immunization in urban slums need to be studied to understand its peculiarities. These peculiarities have the potential to engender policies that ensure optimal immunization for children within urban slums in Nigeria. This study therefore examined the immunization timeliness and completion of infants attending immunization clinics located in urban slums communities in Ibadan, Nigeria between November 2014 and October 2018. It also explored the determinants of immunization completion and timeliness of these infants.

## Materials and methods

### Study design and area

This was a cross sectional study in which data about infant immunization were extracted from the hospital records in the six immunization clinics serving the urban slum communities and the characteristics of the users of the services. This study was conducted in six routine immunization clinics of Primary Healthcare centers (PHCs). These PHCs provide infant immunization services for eight urban slum communities in Ibadan, namely: Òjé, Yemetu, Òkè Aremo, Beere, Ìdí Ògùngún, Basòrun, Aláàádorin and Àténdà. Ibadan is a cosmopolitan city in southwestern Nigeria, and it is the capital of Oyo state. Immunization services in the state are coordinated by the office of the National Primary Health Care Development Agency and the vaccines are being given in both government owned and private health facilities. The activities of the PHCs are coordinated by the Oyo State Ministry of health.

### Selection of primary health centers

Every government owned PHCs which were providing vaccination services in the study communities from 2014 to 2018 were included in this study. There were six PHCs that fulfilled these criteria, and they were all included in the study.

### Data collection and definition of variables

The data from the clinic records of the six PHCs were extracted directly with the aid of a questionnaire. Content validation of the questionnaire was done before the commencement of data collection. The records reviewed covers the four years before the study, from November 2014 to October 2018. These dates were selected for a four-year review period to be completed as the data collection started in November 2018 and ended in December 2018. All the available records were included in the study. The data extracted were the dates of birth, sex, religion, ethnicity, and the date each vaccine was received. All identifiers like the names of the infants and their addresses were not recorded to protect the identity of the infants.

The main outcome variables were infant immunization timeliness and completion. For this study, immunization timeliness was defined as receipt of a vaccine within two weeks of the scheduled date. Immunization completion was defined as receipt of four doses of oral polio vaccine (OPV), a single dose each of Bacille Calmette Guerin (BCG), Hepatitis B, measles and yellow fever vaccine, three doses of the Pentavalent vaccine (made up of Diptheria, Pertussis, Haemophilus influenza B and Hepatitis B antigens and Tetanus toxoid).

### Data analysis

Data were captured and managed with Red Cap hosted at the African Population and Health Research Center, Kenya [21,22]. and exported to SPSS v 25 [23] for analysis. Data were then cleaned and coded to suit the analysis. Timeliness and Completion of immunization was computed using the relevant variables. Univariate analysis was done, and frequencies and proportions were generated for variable such as socio demographics of the infants, timeliness and completeness of immunization. Bar charts and line graphs were also used to represent the distribution of timeliness of each vaccine and the trend across the years studied, respectively. In addition, bivariate analysis was carried out using Chi square test to determine the relationship between selected socio demographic characteristics and timeliness of immunization. All statistical significance was set at 5%.

### Ethical procedure

The study protocol was approved by the Oyo State Ethics Research Committee (AD 13/479/784) and the University of Ibadan/ University College Hospital Institutional Review Board (UI/EC/18/0206). The permission to access the hospital records were also obtained from the Coordinator of the PHC of Ibadan North and Ibadan North East local government areas (under the management of Oyo State Ministry of Health) where the PHCs were located. Identifying data like names and addresses were not included in the data to protect the identity of the infants.

## Results

**Fig 1:**
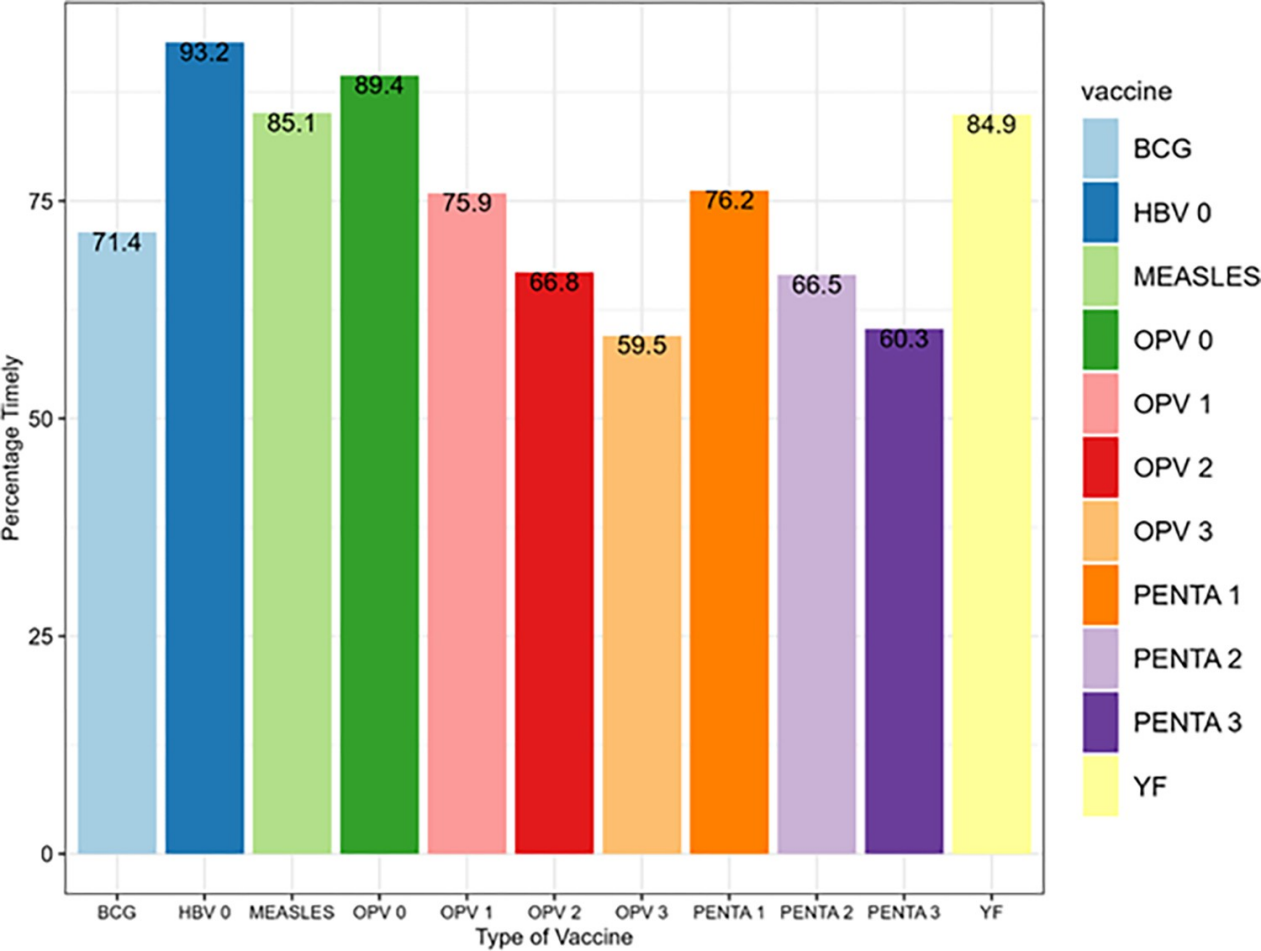
Percentage distribution of infants who received vaccines timely in selected urban slum communities of Ibadan, Nigeria (2014–2019).

The immunization records of 5,934 infants were reviewed, comprising 2,895 (48.8%) females and 3,003 (50.6%) males. The other details of the sociodemographic characteristics of the infants are as shown in Table 1. The percentage of each vaccine that were given on time are shown in Table 2. For each year, the percentage of vaccines given timely reduced from birth till 14 weeks and then increased at nine months. The overall timeliness for each of the vaccines over the four-year period under study is shown in Fig 1. The OPV3 was the least timely of all the vaccines during this period, followed by the Penta3. Among the vaccines taken at birth, it appears that the uptake of BCG was significantly less timely compared with the other vaccines. It also appears that there was a significant reduction in the timeliness of the first Pentavalent vaccine compared with the second dose.

**Table 1:** Socio demographic characteristics of infants whose immunization records were studied for timeliness and completion in selected urban slums of Ibadan, Nigeria.

Selected Sociodemographic characteristics	Frequency	Percentage
**Gender of the infant**		
Male	3003	50.6
Female	2895	48.8
Missing	36	0.5
**Religion**		
Christianity	2873	48.4
Islam	3002	50.6
Missing	59	1.0
**Ethnicity**		
Yoruba	5678	95.7
Hausa	18	0.3
Igbo	182	3.1
Missing	56	0.9

**Table 2:** Timeliness of infant vaccination from records of selected primary health care facilities in urban slum communities of Ibadan, Nigeria (2014–2018).

Vaccine	2014		2015		2016		2017		2018		Total	
	n/N	%	n/N	%	n/N	%	n/N	%	n/N	%	%	n/N
BCG	39/54	72.2	882/1284	68.7	1188/1704	69.7	1214/1663	73.0	506/646	78.3	3821/5351	71.4
OPV 0	38/43	88.4	623/730	85.3	989/1071	92.3	1196/1336	89.5	520/587	88.5	3368/3767	89.4
HBV 0	38/39	97.4	462/500	92.4	914/967	94.5	839/1283	92.3	494/528	93.6	3091/3317	93.2
PENTA 1	23/47	48.9	884/1121	78.9	980/1310	74.8	835/1143	73.4	354/420	84.2	3079/4041	76.2
OPV 1	24/47	51.0	821/1045	78.6	875/1186	73.8	449/1132	73.8	352/422	83.4	2908/3832	75.9
PENTA 2	14/46	30.4	691/997	69.3	707/1085	65.2	446/732	61.3	238/296	80.4	2099/3156	66.5
OPV 2	13/45	28.9	681/975	69.8	694/1056	65.7	303/729	61.2	240/298	80.6	2073/3103	66.8
PENTA 3	7/39	17.9	573/885	64.8	536/888	60.4	303/600	50.5	174/233	74.7	1592/2645	60.2
OPV 3	7/39	17.9	557/856	65.1	491/848	57.9	303/597	50.8	173/232	74.6	1530/2572	59.5
YF	37/38	97.4	484/575	84.2	481/532	90.4	327//425	76.9	37/39	94.9	1366/1609	84.9
Measles	37/38	97.4	486/575	84.5	476/526	90.5	329/426	77.2	36/38	94.8	1364/1603	85.1

BCG—Bacille Calmette Guerin vaccine; HBV—Hepatitis B vaccine; OPV—Oral Polio vaccine; PENTA—Pentavalent vaccine; YF–Yellow fever vaccine.

The timeliness of the yellow fever and measles vaccines also seem to be significantly higher than the third Pentavalent vaccine and OPV3.

**Fig 2:**
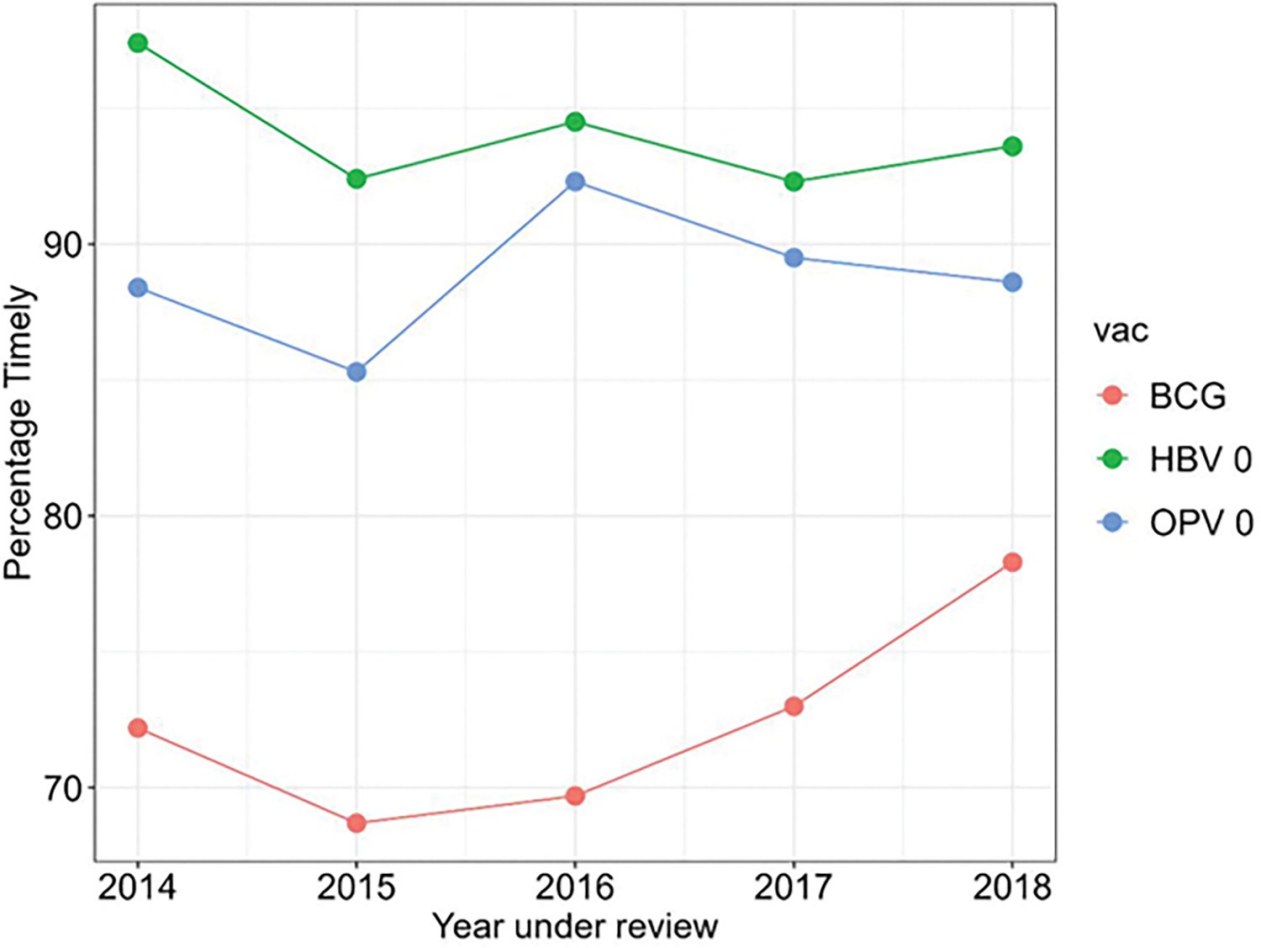
Percentage distribution by year of infants who received timely vaccines at birth in selected urban slum communities of Ibadan, Nigeria.

**Fig 3:**
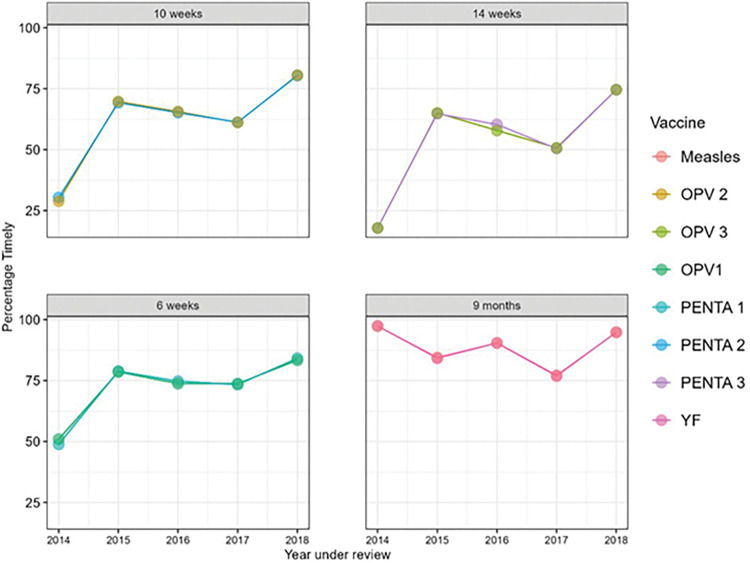
Percentage distribution of infants who received timely vaccines at 6, 10 and 14 weeks, and 9 months of life in selected urban slum communities of Ibadan, Nigeria.

**Fig 4:**
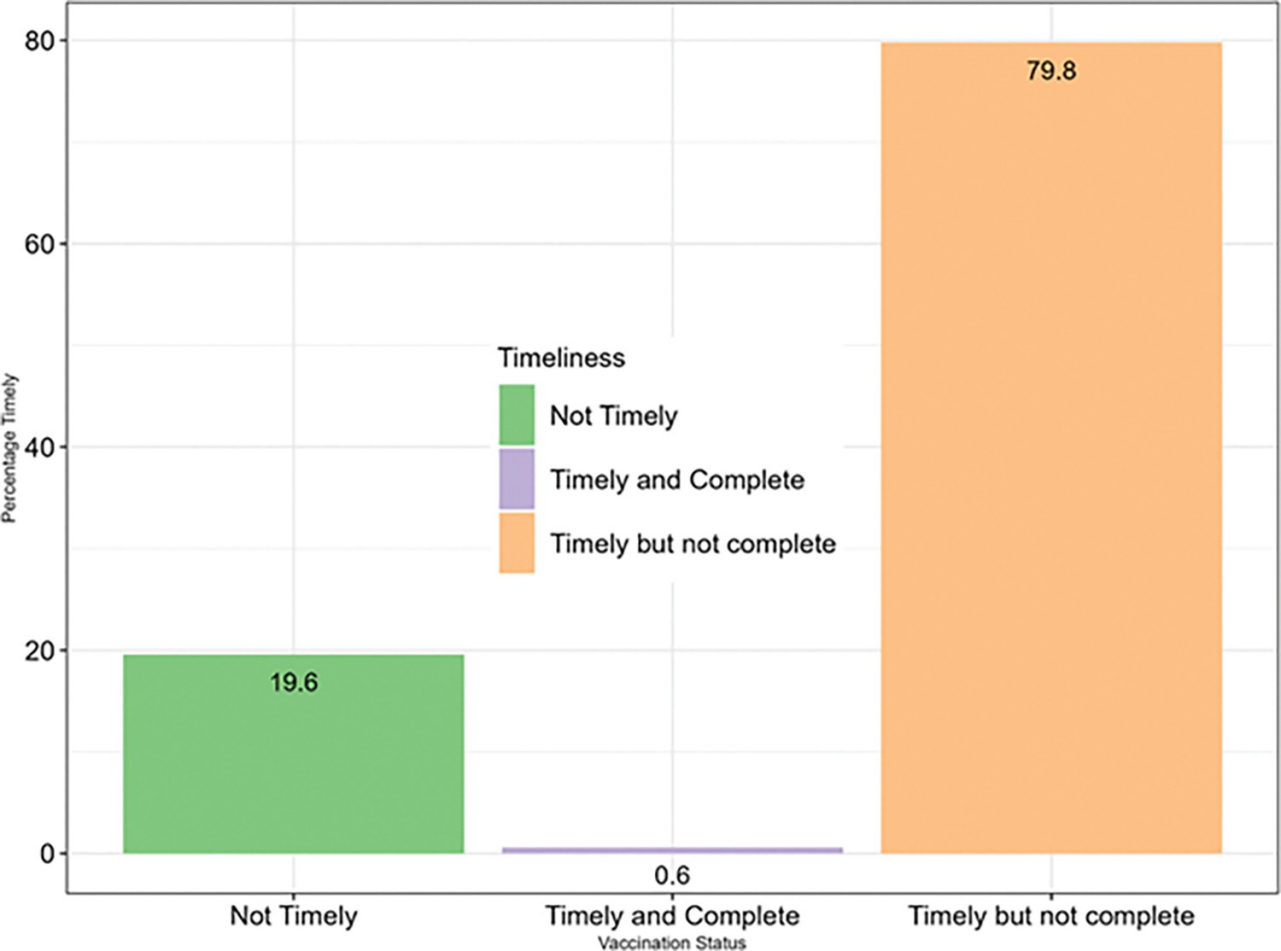
Proportion of infants with timely, incomplete, and complete immunization among infants receiving vaccines at immunization clinic in selected urban slums communities in Ibadan, Nigeria (2014–2018).

**Fig 5:**
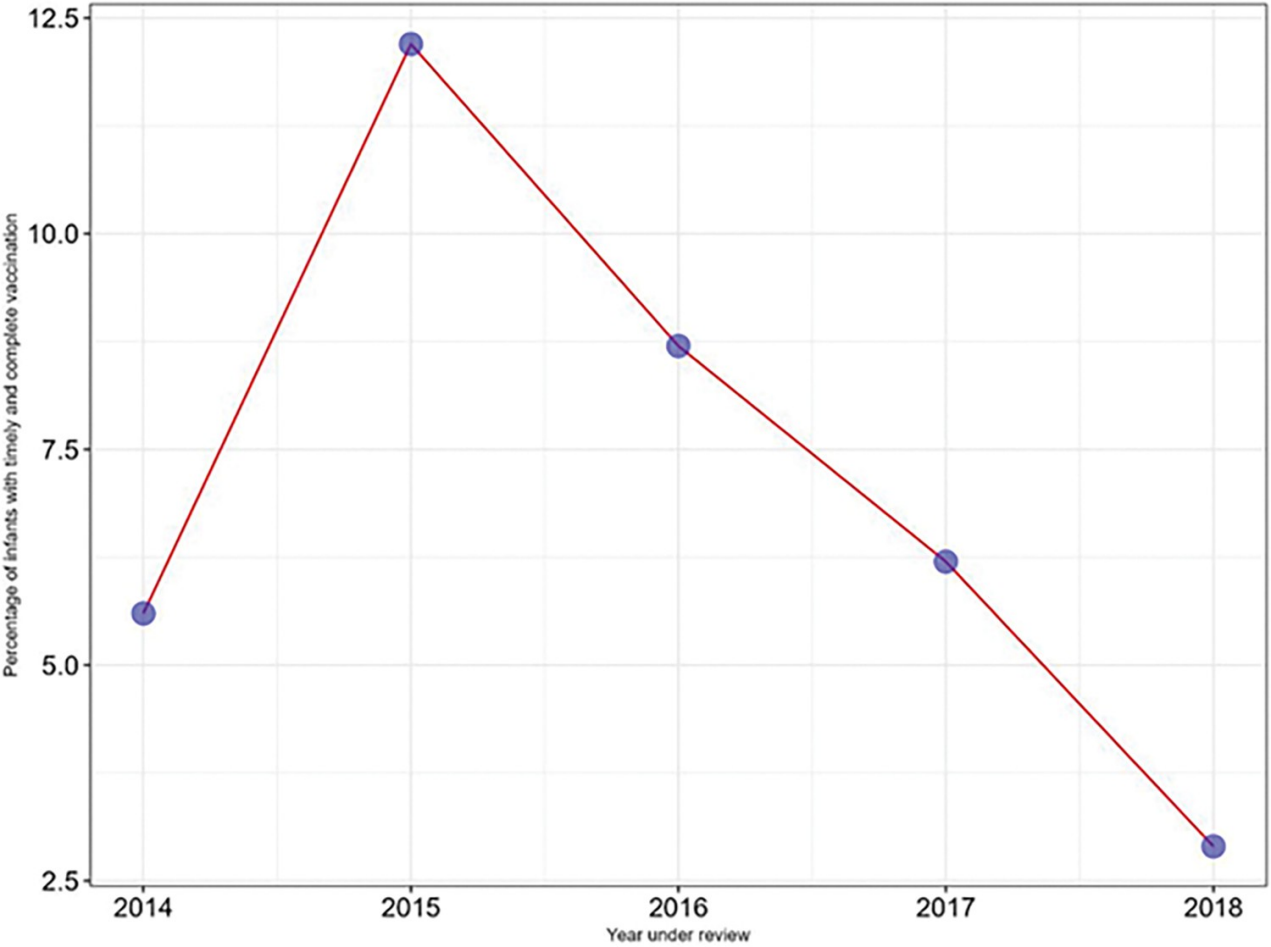
Proportion of infants given timely and complete immunization at selected Primary Health Centers in Urban Slums of Ibadan, Nigeria (2014–2018).

Figs 2 and 3 show the overall timeliness for different groups of vaccines being given at different ages between 2014 and 2018. Fig 2 shows the timeliness of the BCG, HBV0 and OPV0 which were given at birth. BCG was consistently the least timely of the three vaccines while HBV0 was the timeliest. In Fig 3, the timeliness of the pentavalent vaccines and OPV1, OPV2 and OPV3 were inversely proportional to the age of the infants. Also, Fig 3 shows that the timeliness of yellow fever and measles vaccines were least in 2017. Fig 4 shows the overall timeliness and completion of vaccination rates within the years under consideration and only 0.6% of all the infants had both timely and complete vaccinations. Table 3 shows the breakdown of both timeliness and completion of infant vaccinations between 2014 and 2018 and there were more infants with timely but incomplete vaccinations compared with other categories. Fig 5 shows timeliness and completion of infant vaccination by year with the highest proportion of infants with timely and complete vaccination occurring in 2015 (12.2%). This then gradually reduced over the next years and the least timely and completion rate occurred in 2018 (2.9%).

In Table 4, significantly more infants from Muslim families had delayed and incomplete vaccinations compared with those from Christian families. Also, more infants from Christian families had timely but incomplete vaccinations compared with those from Muslim families. The gender and ethnicity of the infants were not significantly associated with timeliness and completion of immunization.

**Table 3:** Immunization Status of infants receiving care at selected Primary Health Centers in Urban Slums in Ibadan, Nigeria (2014–2018).

	Immunization Status					
	Timely			Not Timely		
	Complete	Incomplete	Total	Complete	Incomplete	Total
**2014**	1(2.0)	48(98.0)	49(1.0)	0(0.0)	5(100.0)	5(0.4)
**2015**	19(1.6)	1159(98.4)	1178(24.7)	102(28.4)	257(71.6)	359(30.8)
**2016**	2(0.1)	1490(99.9)	1492(31.3)	59(17.8)	273(82.2)	332(28.5)
**2017**	11(0.7)	1460(99.3)	1471(30.8)	46(12.6)	320(87.4)	366(31.4)
**2018**	1(0.2)	578(99.8)	579(12.1)	1(1.0)	102(99.0)	103(8.8)
**Total**	34(0.7)	4735(99.3)	4769(100.0)	208(17.9)	957(82.1)	1165(100.0)

**Table 4:** Socio-demographic characteristics and association with timeliness and completeness of infant vaccines in selected Primary Health Centers in Urban Slums in Ibadan.

Characteristics	Timely		Not Timely		Total	χ^2^	p-value*
	n	%	n	%			
Gender							
Male	2434	81.1	569	18.9	3003	1.41	0.235
Female	2311	79.8	584	20.2	2895		
Missing	24	66.7	12	33.3	36		
Religion							
Islam	2400	79.9	620	20.1	3020	4.94	0.026
Christianity	2362	81.8	527	18.2	2889		
Missing	40	81.8	15	18.2	55		
Ethnicity							
Yoruba	4567	80.1	1111	19.9	5678	3.26	0.196
Hausa	12	66.7	6	33.3	18		
Igbo	152	83.5	30	16.5	182		
Missing	35	66.0	18	34.0	53		
	**Completed**		**Not completed**				
Gender							
Male	17	0,7	2417	99.3	2434	0.02	0.879
Female	17	0.7	2294	99.3	2311		
Missing	0	0.0	24	100.0	24		
Religion							
Islam	18	0.8	2364	99.2	2382	0.09	0.764
Christianity	16	0.7	2330	99.3	2346		
Missing	0	0.0	40	100.0	40		
Ethnicity							
Yoruba	34	0.7	4533	100.0	4567	1.23	0.540
Hausa	0	0.0	12	100.0	12		
Igbo	0	0.0	152	100.0	152		
Missing	0	0.0	35	100.0	35		

*Missing values were excluded.

## Discussion

Almost all the infants in this study did not complete their immunization schedule during the years under study, although the rate of timeliness for each of the vaccine was improved compared with that reported in earlier studies [24,25]. The pattern of timeliness and completion deserve a detailed investigation as both are important for optimal protection of infants against VPDs. The BCG vaccine was the least timely among the first set of vaccine that were to be given to newborn infants and this has serious implication in the control of tuberculosis, a disease that is associated with poverty and common in the research area. The delay in the administration of the BCG vaccine has been repeatedly associated with the multidose vial policy which makes healthcare workers to insist on having a particular number of infants pooled together before any vial is opened to prevent wastage. This necessitates fixing of specific days of the week for BCG vaccination [26]. The fact that infants must be pooled before BCG vaccine vial could be opened is a plausible reason for the lower timeliness of the BCG vaccine compared to the other vaccines expected to be administered to infants. This can also be responsible for several missed opportunities as some infants may never be presented again for the BCG vaccine. The introduction of single dose vials for BCG may improve the timeliness of this vaccine especially in low dependency clinics since there will be no need to pool infants before vaccines can be administered.

The first dose of the HBV vaccine was the timeliest among the first recommended group of vaccines and this is very important as it is the dose that prevents mother to child transmission of Hepatitis B which is an endemic infection in the research communities. Infection with Hepatitis B in early infancy is associated with the development of chronic infection in which the carrier becomes a reservoir of Hepatitis B infection and the risk of developing hepatocellular carcinoma in future [27]. The pentavalent vaccine timeliness was inversely proportional to the age of the infants just as earlier reported in the literature. The reason for this has been linked with the mothers’ return to their work and distractions with other domestic responsibilities [28]. As a result, mechanisms that serve as reminders are required to ensure the timeliness of these vaccines. Support mechanisms are also important for the mothers so that the responsibility of bringing infants for vaccinations will not rest solely on their shoulders. It is however interesting that the timeliness for yellow fever and measles vaccination was better than that for the pentavalent vaccine series, including the first dose in this series. This is in contrast with earlier reports [25,29,30] and may be due to the inducements (including insecticide treated mosquito nets) that health care workers give to mothers whose infants complete the infant vaccination schedule. This however requires further investigation to understand the factors responsible for the disparity from earlier reports.

Another important finding from this study is that infant vaccination completion rates were abysmally low compared with those reported at the national level for southwest region of Nigeria [31] where the study communities were located and compared with rates reported for urban slums communities in Nigeria [32]. This further corroborates the report that immunization coverage in urban slums is less than that in rural areas in Nigeria as shown by Obanewa et al who revised the immunization coverage data from the Nigerian National Demographic Health Surveys (NDHS) of 2003, 2008 and 2013 [32]. Most families in urban slums are poor and the National Immunization Coverage Survey (NICS) of 2017 showed that only one in ten infants from the poorest families had all the three required pentavalent vaccines [31]. It was however interesting that majority of the infant had timely but incomplete vaccination. This show that there are likely to be challenges or barriers which are discouraging completion of infant vaccination schedule by many infant caregivers in the slum communities under study. The reasons given for noncompletion of infant vaccination in Nigeria from earlier literature include vaccine stock out, lack of awareness of the vaccines the infant require, inconvenient timing and venue of vaccination, and migration of the family [9,33]. These reasons depict caregivers’ inadequate knowledge about infant immunization and unacceptable timing and location of the services. The importance of using the appropriate medium and means to provide correct information about infant immunization to residents of urban slums to drive the demand for these vaccines cannot be over emphasized. There is also the need to diversify the infant vaccination process in Nigeria to improve access to vaccination by providing flexible timing for the service. Mobile clinics may also be better suited in slum communities. The sharp but still suboptimal increase in the infants with timely and complete vaccination in 2015 could be because of likely improved political will for childhood vaccine promotion. Political will have been identified to be essential for the sustenance of successful vaccination programs [34,35]. This is because it facilitates easy access to the necessary resources which are required for the building and continuous improvement of infant vaccination activities.

There was no sex disparity in the timeliness and completion of the vaccinations. Similar findings have been reported by some authors [36,37] but there have been contrasting reports from the literature about sex disparity in infant immunization coverage [38,39] with diverse reasons being given including preference for better care for male infants and the culture of shielding male infants from possible side effects of vaccination in which case, the female infants get disproportionately vaccinated. The reason for this difference requires further investigation in the study communities. There was no religious disparity among the infants with both timely and complete vaccinations. However, more infants from Christian communities also had timely but incomplete vaccinations compared with those from Muslim families. Similar findings were reported from Nigeria and was seen to be more among the slum dwellers. This may be an extension of the belief of Nigerian Muslims about the use of infant vaccinations to spread HIV and to cause sterility with the aim of population control [40]. They may then delay vaccines until they are sure of the vaccine’s safety. Concerted efforts have been made in the past to change this trend, but more work is required to address this disparity.

If data from the urban areas are not disaggregated to show the data from urban slums as previously done in Kenya [39], the variations in the different subgroups in the population will continue to be masked and this will make it impossible to tailor interventions according to their peculiar needs. This population will continue to contribute to the number of infants with suboptimal vaccinations. These include those who will never be immunized, those with delayed vaccination which could result in incompletion of the vaccination schedule, and those with delayed vaccination but completion of the schedule. These last group of infants could develop the targeted infections before vaccination and become contagious to other healthy children. It is therefore imperative that the infant immunization data from Nigerian urban slums are treated separately from other urban areas.

This study has several strengths that need to be highlighted. This is one of the few studies in Nigeria that looked at both timeliness and completion of infant immunization in Nigeria compared with earlier reports which have dwelt mainly on infant immunization coverage without attention to the timeliness of the vaccines despite its importance. Also, the study focused on infant vaccination trend in urban slum communities as against the general urban population, thus unmasking the peculiarities of these communities which is a result of the sociocultural difference from other urban areas. The large data set and the duration of records over a four year period also gives credibility to the findings as it increases the generalizability to other Nigerian urban slum communities.

The study however has some limitations. First, there was limited number of sociodemographic factors which were available in the clinic records, and this limited the number of factors available for consideration for infant vaccination timeliness and completion. Prospective data in the community will have given room for more factors to be considered. The study was also conducted in just one geopolitical zone of Nigeria. There may be peculiarities of other urban slum communities which may be different from those from the study communities.

## Conclusions

In conclusion, although the timeliness of infant vaccination in the studied urban slum communities was better than those from earlier reports, it was still not optimal, and the completion rates were abysmally low. There is a need for targeted interventions to improve both timeliness and completion of infant vaccination to ensure that the infants in these communities are protected from VPDs and by extension, reduce under five mortalities. It is also important that vaccination data from urban areas be disaggregated to show the peculiarity of the different socioeconomic groups which has implications for intervention design.
